# Stretchable and Biodegradable Thermally Expandable Composites with Microfluidics for On‐Demand and Programmable Destruction of Electronics

**DOI:** 10.1002/advs.202505487

**Published:** 2025-06-30

**Authors:** Chan‐Hwi Eom, Won Bae Han, Sungkeun Han, So Jeong Choi, Ikkyo Choi, Jeonguk Kim, Hyewon Cho, Li‐Hyun Kim, Venkata Ramesh Naganaboina, Gwan‐Jin Ko, Tae‐Min Jang, Suk‐Won Hwang

**Affiliations:** ^1^ KU‐KIST Graduate School of Converging Science and Technology Korea University Seoul 02841 Republic of Korea; ^2^ Department of Chemical and Biological Engineering Sookmyung Women's University Seoul 04310 Republic of Korea; ^3^ Department of Electronics and Communication Engineering Amrita School of Engineering Amrita Vishwa Vidyapeetham Amaravati Campus Amaravati Andhra Pradesh 522503 India; ^4^ Display Research Center Samsung Display Gyeonggi‐Do 17113 Republic of Korea; ^5^ Semiconductor R&D Center Samsung Electronics Co., Ltd. Gyeonggi‐Do 18448 Republic of Korea; ^6^ Center of Biomaterials Biomedical Research Institute Korea Institute of Science and Technology (KIST) Seoul 02792 Republic of Korea; ^7^ Department of Integrative Energy Engineering Korea University Seoul 02841 Republic of Korea

**Keywords:** biodegradable, destruction system, drug‐delivery, thermally expandable composite, transient electronics

## Abstract

The lifespan of the transient electronic system can be determined in advance (i.e., predefined) or controlled via on‐demand and programmable approaches using a diverse range of principles. However, in most cases, dissolution or disappearance requires an aqueous solution and is only possible for the entire system, not for specific or targeted components. Here, a soft, stretchable, thermally expandable system is introduced for precise, localized, on‐demand deactivation or destruction of electronic systems. The incorporation of thermal expansion particles into a polymer matrix produces soft, resilient composites that generate substantial thermo‐mechanical forces at a predefined temperature, enabling the direct collapse of electronic devices. Integration with multichannel microfluidics and wireless systems creates a vanishing, self‐destructive optoelectronic system and bio‐safe drug delivery vehicle for frequency‐based selective release, demonstrating the broad potential of this approach in the fields of defense/security and biomedical devices as well as other envisioned areas.

## Graphical abstract

Soft, stretchable, thermally expandable composites that enable tunable, mechanically driven, on‐demand destruction of transient electronics are developed. Integrated with microfluidics and wireless circuits, the composites facilitate precise, selective device collapse and frequency‐controlled drug release, highlighting the broad potential in implantable biomedical devices, sustainable electronics, and hardware security systems.

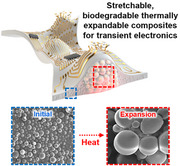

## 1 Introduction

Unlike conventional, modern electronic systems designed for long‐lasting and reliable operation without malfunction, degradable and biosafe materials‐based transient systems aim to perform temporary functions and then physically dissolve or disappear. This unique feature is useful for unprecedented applications in implantable biomedical devices,^[^ ^1^, ^2^, ^3^ ^]^ environmentally sustainable electronics,^[^ ^4^, ^5^ ^]^ and hardware security systems.^[^ ^6^, ^7^, ^8^ ^]^ Since the transience of electronics primarily relies on reactive dissolution by hydrolysis of constituent materials,^[^ ^9^, ^10^ ^]^ a wide range of encapsulation or passivation methods were proposed to manipulate operational lifetimes.^[^ ^11^, ^12^, ^13^ ^]^ One typical method is passive transience, where the device lifetime is predetermined at early stages.^[^ ^3^, ^14^, ^15^, ^16^ ^]^ The other is to utilize external stimuli‐responsive materials – capable of decomposition or disintegration upon exposure to heat, light, or pH levels – for triggered or programmable control over degradation.^[^ ^17^, ^18^, ^19^, ^20^, ^21^ ^]^ Although the latter provided a platform for more effective regulation through a variety of factors, both formats require undergoing a chemical reaction with an aqueous medium, imposing constraints on the selective dissolution of local, specific components, particularly in solid‐state electronic devices.^[^ ^22^, ^23^, ^24^ ^]^ To overcome the limitations of material‐driven degradation, physical/mechanical deformations as stimulants provide non‐aqueous, tunable transience. One of the exampled approaches leveraged heat‐activated expansion to induce mechanical collapse of neighboring electronic components, enabling on‐demand, remotely triggered transiency with rapid activation.^[^ ^22^ ^]^ A similar idea, yet with a microfluidic channel system, offered the ability to control the flow of etchants for selective and complete dissolution of targeted components.^[^ ^25^ ^]^ Another study proposed a physicochemical reaction using non‐toxic chemical agents to generate carbon dioxide bubbles, driving the biocompatible disintegration of multifunctional transient systems.^[^ ^8^ ^]^

Here, we introduce soft, stretchable, thermally expandable components that enable on‐demand, triggered mechanical destruction or collapse of electronic systems. The incorporation of thermally expandable particles (TEPs) into a polymer matrix yields thermal expansion composites (TECs) with tunable behaviors, and comprehensive thermomechanical characterization explores the mechanical resilience and cyclic stability, and force‐relevant factors. Integration of TECs with multichannel microfluidics and wireless circuits enables to formation of a microscale vanishing, self‐destructive optoelectronic system and drug delivery platform for frequency‐selective drug release with safe disposal. This approach offers minimal complexity, wireless programmability, and spatial selectivity compared to existing thermal expansion‐based methods (Table S1, Supporting Information), highlighting the broad potential in hardware‐security and biomedical applications.

## 2 Results and Discussion

### 2.1 TEC for Self‐Destructive Transient Electronics

**Figure 1:**
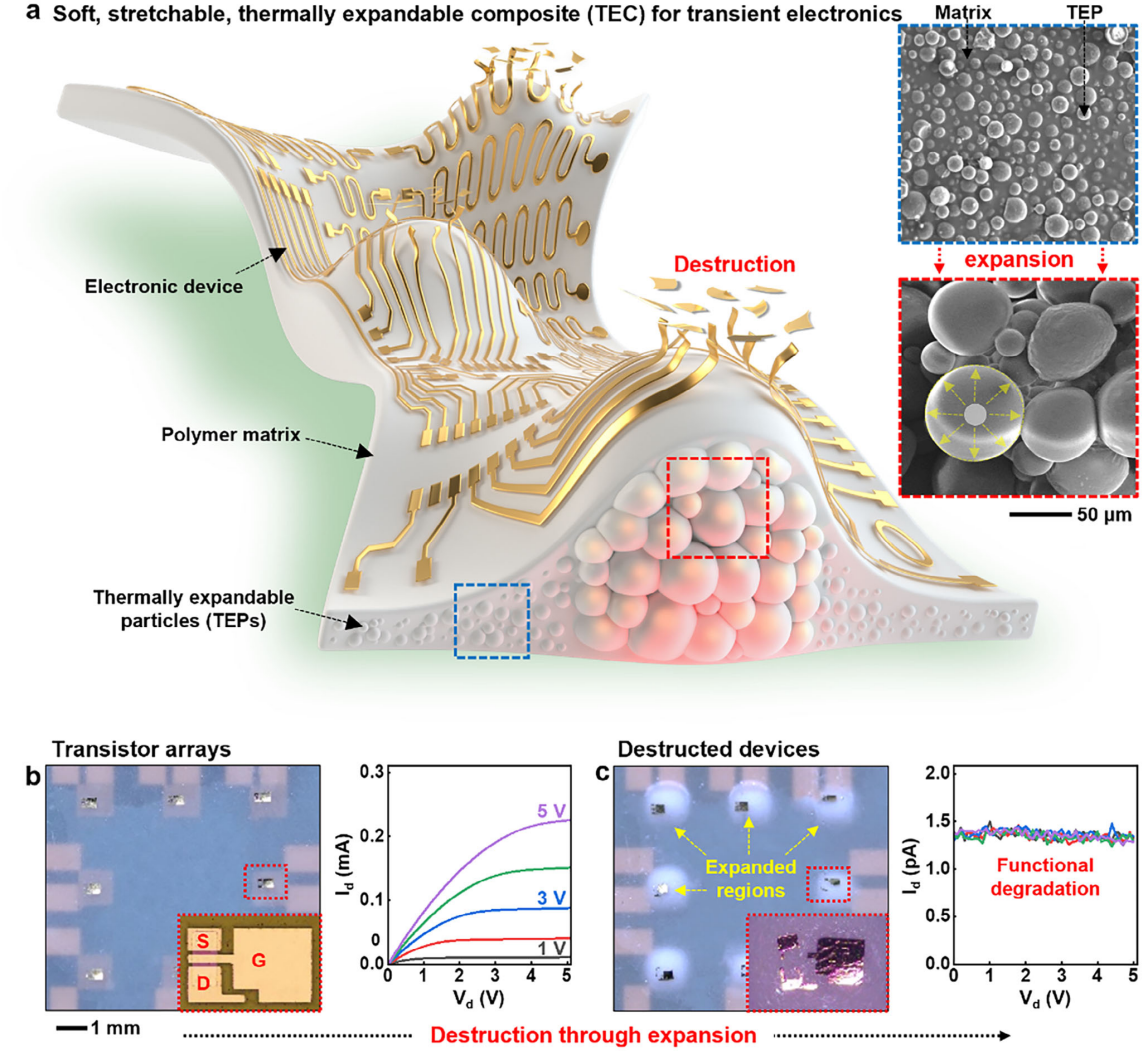
Soft, stretchable, TEC for transient electronics. a) Illustration of a soft, stretchable transient electronic system capable of on‐demand, self‐destruction through heat‐induced expansion of a TEC substrate composed of TEPs embedded in a polymer matrix, and SEM images of a TEC film before and after expansion in the inset. b,c) Optical images and *I*–*V* characteristics of n‐channel metal‐oxide‐semiconductor field effect transistors (n‐MOSFETs; channel length and width, 20 and 100 µm) arrays with silicon nanomembranes (Si NMs)‐based on a TEC substrate before (b) and after (c) expansion of the substrates.

**Figure** **1**a illustrates a soft, stretchable, self‐destructive electronic system based on a TEC comprising a soft polymer matrix embedded with TEPs – gas‐encapsulating polymer shells that undergo substantial volumetric expansion when heated above a threshold temperature. The polymer shells softened and internal gas pressure increased, causing the TEPs to expand ≈60 times the original volumes,^[^ ^26^ ^]^ which induced rapid structural disintegration of overlying electronic components, ultimately leading to device malfunctions. As a proof‐of‐concept, Figures 1b (before) and 1c (after) provide a set of images (left) and characteristics (right) of representative electronic devices before and after physical breakdown through the TEC expansion. The devices consisted of an array of n‐channel metal‐oxide‐semiconductor field‐effect transistors (n‐MOSFETs) on a TEC film, integrated with copper (Cu, 200 nm)‐based microscale heaters (µ‐heaters) array placed on the corresponding locations underneath (Figure S1, Supporting Information). When the µ‐heaters were activated to generate thermal energy in desired positions, a large volumetric expansion of the TEC film mechanically burst the transistor array with deteriorations in electrical functions. For isolated and focused heating at target regions, flame resistant FR‐4 (500 µm thick) was employed as the substrate for the µ‐heaters array unless noted otherwise, due to its relatively low thermal conductivity and mechanical flexibility^[^ ^27^, ^28^ ^]^ (Figure S2, Supporting Information).

### 2.2 Mechanical and Thermal Expansion Property of TECs

**Figure 2:**
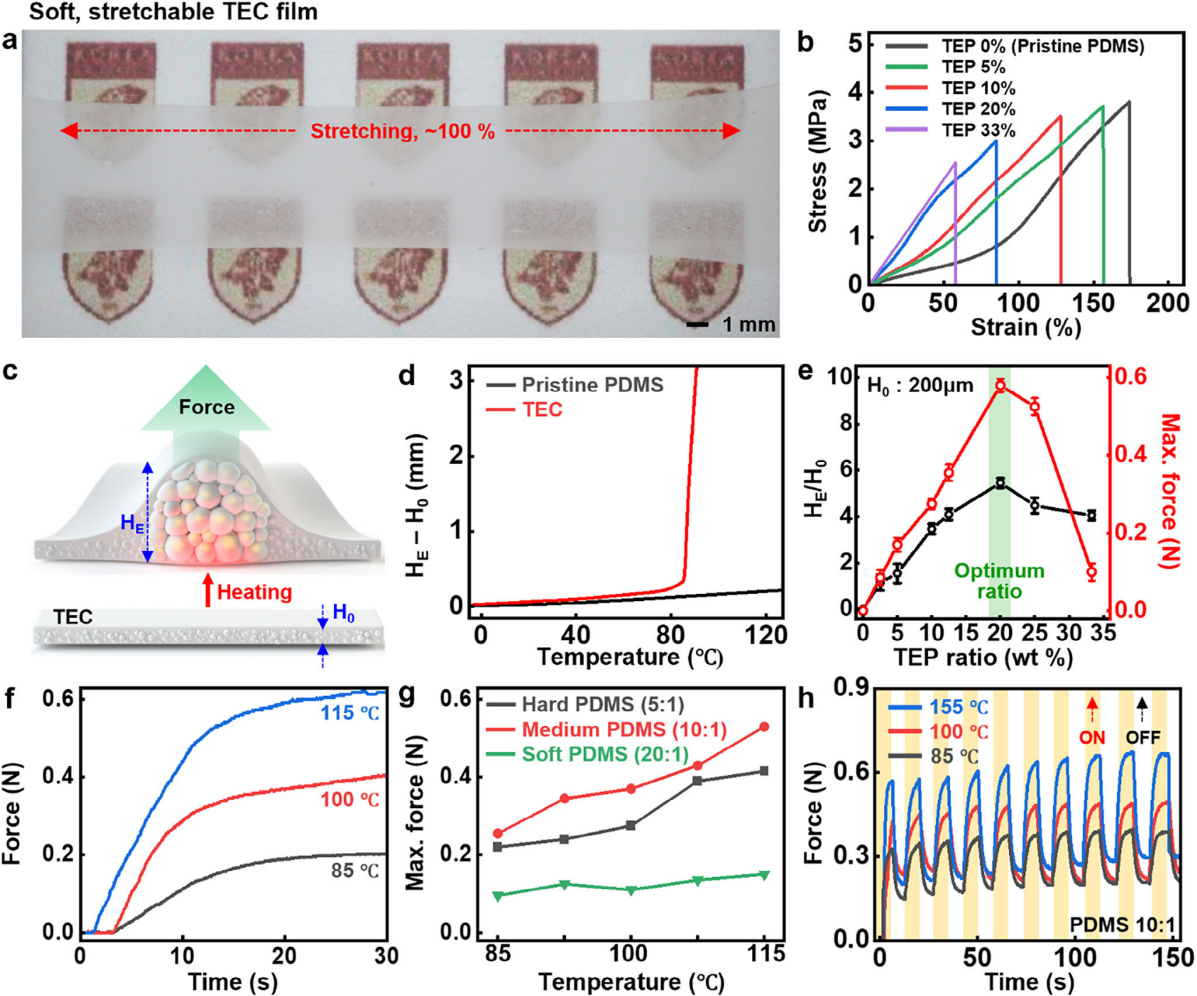
Characterization of soft, stretchable TEC. a) Optical image of a TEC film (10% TEP in PDMS, 200 µm thick) under a linear strain of ≈100%. b) Stress‐strain curves of TEC films with varying TEP contents (0%, 5%, 10%, 20%, and 33%). c) Schematics of the force generated during thermal expansion of a TEC film from initial (H_0_) to expanded thicknesses (H_E_). d) Changes in thicknesses of pristine PDMS and a TEC film (TEP, 20%) over a temperature range from −5 °C to 125 °C, with CTEs of 284 and 65757 µm m^−1^·°C^−1^, respectively. e) Ratios of expanded to initial thickness (H_E_/H_0_) and maximum thermal expansion forces of TEC films with varying TEP contents (0–33%). All data are presented as mean ± SD (n = 10). f) Temporal changes in thermal expansion forces of a TEC film at different temperatures of 85 , 100, and 115 °C. g) Maximum thermal expansion forces of TEC films fabricated with different PDMS crosslinking ratios (5:1, 10:1, and 20:1 in base to curing agent ratio). h) Cyclic mechanical behaviors of TEC films upon repetitive heating at 85, 100 , and 115 °C.

Among the tested TEP candidates (Expancel 031 DU 40, 051 DU 40, and 053 DU 40), 031 DU 40 for experiments was selected for the following experiments due to its lowest onset temperature (≈85 °C) and highest coefficient of thermal expansion (CTE) (Figure S8, Supporting Information). **Figure** **2**a illustrates a TEC film composed of 10 wt.% TEPs embedded in a polydimethylsiloxane (PDMS) matrix with a 10:1 base‐to‐curing agent ratio, under a tensile strain of ≈100%. This elastic behavior was consistently retained across TEP concentrations of 0 ≈20%, although the elasticity (75–175%) decreased as the amount of particles increased (Figure 2b). An increase in TEP concentrations raised the Young's modulus of TEC while slightly decreasing the fracture strain, attributed to the higher modulus of TEPs compared to PDMS. Figure 2c describes the thermal expansion mechanism of TEPs, which induces a thickness change of TEC from the initial (H_0_) to the expanded (H_E_) state, thereby generating a vertical expansion force. As shown in Figure 2d, a TEC with 20 wt.% TEPs exhibited a rapid and dramatic change of thickness (H_E_ – H_0_) at ≈85 °C, with a CTE of ≈65 000 µm m^−1^·°C^−1^. In contrast, pristine PDMS without TEPs showed negligible changes of thickness over 0–130 °C, with a much lower CTE (284 µm m^−1^·°C^−1^). Figure 2e presents ratios of the thickness change (H_E_/H_0_) and maximum force generated during thermal expansion at various TEP concentrations. All data represent mean ± standard deviation (n = 10). The optimal TEP content (20 wt.%) achieved the highest thickness change ratio (≈5) and maximum force (≈0.6 N). Lower TEP concentrations provided insufficient particles for significant mechanical responses, while higher concentrations caused particle agglomeration and thus ineffective thermal expansion.^[^ ^29^ ^]^ The expansion force can be controlled with applied temperatures, as higher temperatures activate more TEPs and generate greater forces. When the system was given a heat of 115 °C, rapid and large increases in the maximum force were achieved compared to 85 and 100 °C (Figure 2f; Figure S3, Supporting Information). Mechanical properties of the polymer matrix in the composite are also a determination factor (Figure 2g). TECs with PDMS (10:1) exhibited the highest forces, while stiffer (5:1) and softer (20:1) PDMS suppressed and absorbed particle expansion energy, i.e., reduction of output forces. Figure 2h displays the cyclic thermal expansion behavior under pulsed heating at 85 °C, 100 °C, and 115 °C over 10 cycles. The TECs showed generally stable force generation and reversible behavior with slight hysteresis, confirming that mechanical integrity was maintained under repeated actuation.

### 2.3 Microfluidic Channel‐Integrated On‐Demand Destruction System

**Figure 3:**
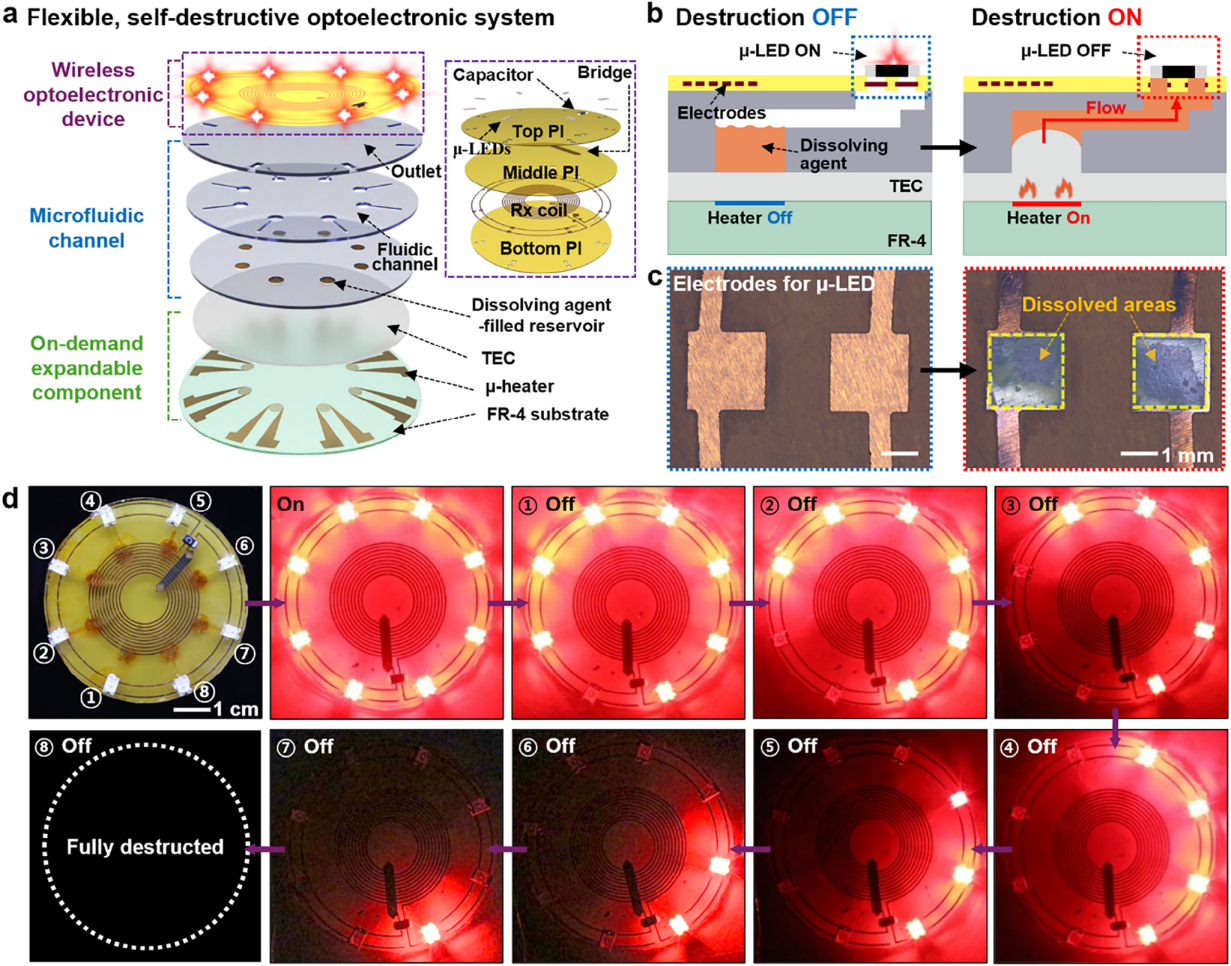
Application of TEC to a self‐destructive electronic system. a) Exploded view of a flexible, self‐destructive optoelectronic system featuring a wireless optoelectronic device, a microfluidic channel, and an on‐demand expandable component. The optoelectronic device includes a microscale light‐emitting diode (µ‐LED) array on a Cu/polyimide (PI)‐based wireless power receiver; the microfluidic system comprises a PDMS‐based outlet layer (top), a fluidic channel (middle), and a reservoir layer containing dissolving agents (bottom); and the expandable component consisted of a TEC layer on a µ‐heater array supported by FR‐4 substrate. b) Destruction mechanism of the optoelectronic system. Local thermal expansion of the TEC layer by µ‐heaters induced the flow of dissolving agents through the microfluidic channel, leading to the degradation of Cu electrodes and subsequent device malfunction. c) Optical images of electrodes before and after destruction. d) Sequential images of the on‐demand destruction process of the wireless optoelectronic system.

Thermal expansion composites can be connected to diverse tools or systems to evolve into more sophisticated and controlled forms. **Figure** **3**a presents a wireless, flexible optoelectronic system designed to demonstrate on‐demand destruction through microfluidic channels assembled with thermally expandable components. This self‐destructive system comprised three main parts (from top to bottom): i) a wireless, optoelectronic device integrated with micro‐light‐emitting diodes (µ‐LEDs); ii) a PDMS‐based microfluidic system involving outlets, fluidic channels, and reservoirs; and iii) expandable TEC components with µ‐heaters. The detailed fabrication process and measured resonant frequency of the optoelectronic device appear in Figures S4 and S5 (Supporting Information), respectively. Figure 3b provides a cross‐sectional illustration of the destruction mechanism. Localized heating caused expansion of the TEC, generating mechanical force that allowed the dissolving agent stored in the reservoirs to flow through the channels and reach the outlets that were strategically connected to electrodes for the µ‐LEDs. Then, the dissolving agent chemically interacted with the electrodes, degrading electrical interconnections and terminating device functionality. Figure 3c displays images of the electrodes before (left) and after (right) the complete dissolution through such a destruction process. Figure 3d demonstrates the capability of the on‐demand destruction system. Sequential activation of µ‐heaters in a designated order (1–8) led to the termination of µ‐LEDs in a well‐defined, independent manner. This highlights the potential of the microfluidic channel‐integrated system for remote and precise collapses of electronic devices across various applications.

### 2.4 Soft, Biodegradable, Wireless Drug Delivery System

**Figure 4:**
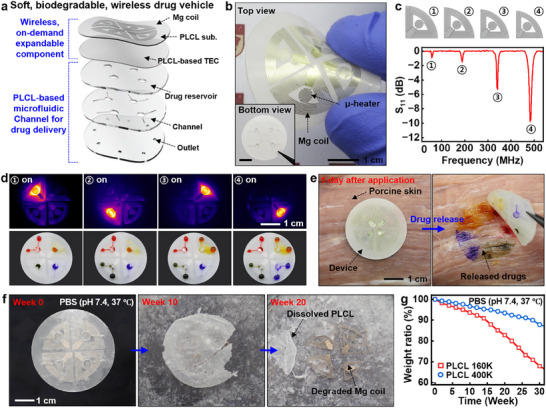
Soft, biodegradable, wireless microfluidic system for on‐demand, controlled release of multi‐therapeutic agents. a) Exploded view of an on‐demand biodegradable drug vehicle, comprising a PLCL substrate integrated with Mg wireless heaters operating at different frequencies (top), a PLCL‐based TEC layer (top middle), a drug reservoir layer (middle), a fluidic channel layer (bottom middle), and an outlet layer (bottom). b) Optical images of the biodegradable wireless drug delivery system. c) The design approach of wireless coils with different resonant frequencies. d) Infrared (IR) thermograms (top) and optical images (bottom) show sequential activation of the wireless heaters (1–4) and corresponding drug release. e) Images of the drug delivery system attached to porcine tissues, captured before (left) and after (right) drug activation. f) Photographs at various dissolution stages of the drug delivery system when immersed in phosphate buffer solution (PBS; pH 7.4) at 37 °C. g) Dissolution behaviors of devices fabricated using PLCLs with different molecular weights (M_n_) of 160 and 400k.

Such expandable features can be utilized for a customized drug‐eluting system. **Figure** **4**a presents a soft, biodegradable, wireless drug delivery vehicle using dissolvable magnesium (Mg) foil for µ‐heater‐integrated wireless radio frequency (RF) coils on a biodegradable elastomer, poly(lactide‐co‐ɛ‐caprolactone) (PLCL), and microfluidics‐included TEC components. This system can release different types of drugs loaded in separate reservoirs via individual activations of wireless µ‐heaters with clearly distinct frequencies. Figure 4b shows a top view of a representative fabricated system, with a bottom view in the inset. The Mg RF coils were designed with different configurations, resulting in distinct resonant frequencies of ≈50, 200, 350, and 500 MHz (Figure 4c). This feature enabled independent operation of each µ‐heater without mutual interference, as confirmed by the sequential activation of the heaters at their respective resonant frequencies and consequent drug release in the targeted areas (Figure 4d). Thermal expansion behaviors of PLCL‐based TECs appear in Figure S9 (Supporting Information). To further quantify and control the heating behavior of the wireless heaters, we measured the induced voltage at each Rx coil under varying Tx–Rx distances. The results revealed a steep decrease in voltage with increasing distance, consistent with magnetic coupling decay. In addition, a voltage–temperature calibration curve was obtained for the microheater to determine the precise actuation voltage required for thermal expansion (Figure S10, Supporting Information). Figure 4e demonstrates the drug delivery capability of the system on porcine skin. Without µ‐heater activation, the device exhibited no unintended drug release during day‐long implantation and even under mild shaking or handling. Such stability could be attributed to the capillary forces within the microfluidic channel, which significantly exceeded gravitational effects.^[^ ^30^ ^]^ In contrast, activation of the µ‐heaters successfully triggered the release of all stored drugs into the tissue. We note that the low thermal conductivity of PLCL (0.158 W m^−1^·K^−1^) minimized heat transfer (Figure S6, Supporting Information), preventing potential burns or damages to surrounding skin or tissues during operation.

Figure 4f,g present degradation behaviors of the drug delivery system in PBS(pH 7.4) at 37 °C. The device retained its structural and functional integrity for up to 2 weeks of immersion (Figure S7, Supporting Information) and underwent significant degradation over 20 weeks via hydrolysis, leaving only minimal PLCL and Mg residues. The device lifespan can be tailored by adjusting the molecular weight of PLCL, enabling long‐term functional support for implantable/biomedical and eco‐friendly applications (Figure 4g).

## 3 Conclusion

The concepts, materials, and system applications reported here illustrate the volumetric expansion of TECs as an effective strategy for the rapid and localized destruction of soft, stretchable, transient electronics. Mechanical and thermal analysis revealed that TEP‐to‐polymer composition significantly influences thermal expansion behavior and mechanical resilience, where 20 wt.% TEPs achieved the highest expansion ratio and force generation for abrupt structural collapse. Microfluidic channels‐integrated optoelectronic system validated the potential of TECs for on‐demand and selective electronic destruction. The integration of PLCL‐based TECs with Mg‐based heaters and RF coils enabled a soft, biodegradable drug delivery system, allowing for frequency‐selective drug release with controlled degradation over 20 weeks.

## 4 Experimental Section

### Preparation of PDMS‐Based TEC Solution and on‐Demand Expandable Component

PDMS‐based TEC films were prepared using PDMS (Sylgard 184, Dow Corning, USA) at various base to curing agent ratios of 5:1, 10:1, and 20:1, and TEPs (Expancel 031 DU 40, Nouryon, USA) at varying concentrations of 0, 2.5, 5, 10, 20, 25, and 33 wt.%. The resulting PDMS/TEP mixtures were spin‐coated onto micro‐patterned heater arrays on FR‐4 substrate to achieve a uniform film, followed by curing in a convection oven at 60 °C for 12 h to solidify the TEC structure.

### Fabrication of *n*‐Type Si‐NMs‐Based n‐MOSFET Device Array on TEC

Fabrication began with a spin coating of PI (Sigma–Aldrich, USA) on a silicon oxide wafer. N‐type Si‐NMs (300 nm) were then transferred onto the PI‐coated SiO_2_ wafer. The doped Si‐NMs were patterned using sulfur hexafluoride (SF_6_)‐based reactive ion etching (RIE, JV19RIE‐8AP, JVAC) to define active areas. A 100 nm thick layer of SiO_2_ was deposited by plasma enhanced chemical vapor deposition (PECVD, Plasmalab 800 plus, Oxford, UK) and patterned. Buffered oxide etchant (BOE, 6:1, Sigma–Aldrich) was employed to selectively open the oxide layer at source and drain contact regions, allowing for electrical connections. Subsequently, a 250 nm thick gold layer was deposited by an e‐beam evaporator (KVE‐T5292, Korea Vacuum Co.) and lithographically patterned to form the source and drain electrodes. The patterned *n*‐type metal‐oxide‐semiconductor (NMOS) device array was lifted off from the silicon oxide wafer using UV release tapes and transferred onto the TEC. Finally, the PI layer was etched away using oxygen reactive ion etching (O_2_ RIE, JVRIE17‐8TM, JVAC) to complete the fabrication of the system.

### Fabrication of a Flexible, Self‐Destructive Optoelectronic System—Wireless Optoelectronic Device

Fabrication began by attaching a Cu foil (thickness: 5 µm, Nilaco, Japan) to a glass substrate using PI tape. A PI solution (Sigma–Aldrich, USA) was spin‐coated over the foil and cured to form a bottom PI layer, followed by transfer‐printing of the Cu/PI bilayer onto PDMS‐coated glass substrates. Photolithography was employed to pattern the Cu foil into a receiver (Rx) coil structure, and a middle layer of PI was subsequently created over the patterned structure, and dry etching with O_2_ RIE opened the contact regions for an electrical bridge. A 2 µm‐thick Cu film was then deposited by sputtering (KVS‐2004, Korea Vacuum Tech) and patterned using a lift‐off process to define the conductive bridge. An additional PI layer (top PI) with openings to contacts for µ‐LEDs and a capacitor was formed for encapsulation. After removal from the PDMS substrate, the whole samples were turned over to open regions on the bottom PI layer, where the dissolving agents (Cu ETCH APS‐100, YMS tech, South Korea) from reservoirs flow through channels. Finally, µ‐LEDs and a capacitor were attached to the top of the opened areas using low‐temperature conductive epoxy (≈70 °C, MG Chemicals) to complete the fabrication.

### Fabrication of a Flexible, Self‐Destructive Optoelectronic System—Microfluidic Channel

PDMS solution was poured into pre‐fabricated epoxy (SU‐8 100, MicroChem Inc.) molds on a silicon wafer and cured in a convection oven at 60 °C for 12 h. After curing, the PDMS films were carefully peeled to create layers of reservoir, fluidic channel, and outlet, and these layers were bonded via O_2_ plasma treatment to complete the microfluidic system.

### Fabrication of a Flexible, Self‐Destructive Optoelectronic System—On‐Demand Expandable Component

The fabrication process followed the same steps as described above for PDMS‐based TEC.

### Fabrication of Wireless, Biodegradable On‐Demand Drug Delivery System

A biodegradable, elastic polymer – PLCL – was dissolved in ethyl acetate (EA, Sigma‐Aldrich, USA) at a concentration of 25 w/v%, and the solution was poured into a silicone mold and dried at 50 °C for 12 h to prepare both neat PLCL and PLCL‐based TEC films with incorporating 20 wt.% of TEPs. For a soft, biodegradable microfluidic channel, neat PLCL films were laser‐cut into shapes for the drug reservoir, fluidic channel, and outlet, and these layers were precisely aligned and chemically bonded by applying a small amount of EA at the interfaces and curing at 60 °C for 2 h. For wireless, on‐demand, expandable components, Mg foil (30 µm) attached to a PDMS substrate was laser‐cut into four different design layouts and transfer‐printed onto the PLCL‐based TEC films. Then, a dielectric PLCL layer (≈50 µm) and Mg foil were sequentially laminated over the structures to establish electrical connections, followed by deposition of an additional PLCL layer (≈100 µm) for encapsulation. Finally, the TEC layer of the wireless, expandable component was bonded to the reservoir layer of the PLCL‐based microfluidic channel.

### Characterization of Morphological/Mechanical Properties of TEC

TEPs within the TEC were examined using a scanning electron microscope (SEM‐4300, Hitachi). Tension and force tests were performed to evaluate the mechanical properties of the TEC using a force tester (ESM303, MARK‐10, USA). For tensile testing, dog‐bone‐shaped specimens were prepared with a length of 7 mm, a width of 5 mm, and a thickness of 0.2 mm. It stretched at a rate of 6 mm min^−1^. Force measurements were conducted using a force tester equipped with a convex tip, enabling precise application and localized measurement of forces.

### Electrical Signal Measurement and Wireless Power Transfer Characterization

For the NMOS device, the output voltages before and after destruction were measured using a probe station (M6VC, Keithley 4200). For the wireless device, RF signals were generated using a waveform generator (E4432B, HP, USA) and amplified by an RF amplifier (M75, Instruments for Industry, USA) before being transmitted to the device's receiving antenna. The scattering parameter (S_11_) – the reflection coefficient, was obtained using a vector network analyzer (MS 2024A, VNA Master, JAPAN) to characterize the RF signal behavior and ensure optimal transmission resonant frequencies.

### Degradation Analysis

Samples were immersed in PBS (pH 7.4) at room temperature for a 20‐week period to evaluate their degradation behavior. Each week, the samples were removed from PBS solution, rinsed with deionized (DI) water, and dried using a freeze dryer (TFD 8503, IlShinBioBase, South Korea) to ensure complete removal of moisture. The weight ratio (%) was calculated using the formula: weight ratio (%) = W_a_ / W_b_ x 100, where W_b_ denotes the weight of the sample before immersion, and W_a_ indicates its weight after immersion

## Conflict of Interest

The authors declare no conflict of interest.

## Supporting information



Supporting Information

## Data Availability

The data that support the findings of this study are available from the corresponding author upon reasonable request.

## References

[1] W. B. Han , G.‐J. Ko , K.‐G. Lee , D. Kim , J. H. Lee , S. M. Yang , D.‐J. Kim , J.‐W. Shin , T.‐M. Jang , S. Han , H. Zhou , H. Kang , J. H. Lim , K. Rajaram , H. Cheng , Y.‐D. Park , S. H. Kim , S.‐W. Hwang , Nat. Commun. 2023, 14, 2263. 37081012 10.1038/s41467-023-38040-4 PMC10119106

[2] T.‐M. Jang , W. B. Han , S. Han , A. Dutta , J. H. Lim , T. Kim , B. H. Lim , G.‐J. Ko , J.‐W. Shin , R. Kaveti , H. Kang , C.‐H. Eom , S. J. Choi , A. J. Bandodkar , K.‐S. Lee , E. Park , H. Cheng , W.‐H. Yeo , S.‐W. Hwang , Sci. Adv. 2024, 10, adp9818. 10.1126/sciadv.adp9818 PMC11373598 39231226

[3] J. H. Lim , W. B. Han , T.‐M. Jang , G.‐J. Ko , J.‐W. Shin , S. Han , H. Kang , C.‐H. Eom , S. J. Choi , K. Rajaram , A. J. Bandodkar , W.‐H. Yeo , S.‐W. Hwang , Biosens. Bioelectron. 2024, 254, 116222. 38518560 10.1016/j.bios.2024.116222

[4] M. Irimia‐Vladu , E. D. Głowacki , G. Voss , S. Bauer , N. S. Sariciftci , Mater. Today 2012, 15, 340.

[5] T. Mekonnen , P. Mussone , H. Khalil , D. Bressler , J. Mater. Chem. A Mater. Energy Sustain. 2013, 1, 13379.

[6] H. Bae , B.‐H. Lee , D. Lee , M.‐L. Seol , D. Kim , J.‐W. Han , C.‐K. Kim , S.‐B. Jeon , D. Ahn , S.‐J. Park , J.‐Y. Park , Y.‐K. Choi , Sci. Rep. 2016, 6, 38324. 27917910 10.1038/srep38324 PMC5137035

[7] Y. Gao , Sci. Rep. 2017, 7, 45391. 28350003 10.1038/srep45391 PMC5368977

[8] J.‐W. Shin , J. Chan Choe , J. H. Lee , W. B. Han , T.‐M. Jang , G.‐J. Ko , S. M. Yang , Y.‐G. Kim , J. Joo , B. H. Lim , E. Park , S.‐W. Hwang , ACS Nano 2021, 15, 19310. 34843199 10.1021/acsnano.1c05463

[9] Y. Zhang , G. Lee , S. Li , Z. Hu , K. Zhao , J. A. Rogers , Chem. Rev. 2023, 123, 11722. 37729090 10.1021/acs.chemrev.3c00408

[10] M. Monisha , S. Agarwala , Mater. Sci. Add. Manuf. 2022, 1, 15.

[11] G.‐J. Ko , H. Kang , W. B. Han , A. Dutta , J.‐W. Shin , T.‐M. Jang , S. Han , J. H. Lim , C.‐H. Eom , S. J. Choi , Y. Ryu , W.‐H. Yeo , H. Cheng , S.‐W. Hwang , Adv. Funct. Mater. 2024, 34, 2403427.

[12] W. B. Han , S.‐W. Hwang , W.‐H. Yeo , Flex. Print. Electron. 2024, 9, 033001.

[13] W. B. Han , G.‐J. Ko , S. M. Yang , H. Kang , J. H. Lee , J.‐W. Shin , T.‐M. Jang , S. Han , D.‐J. Kim , J. H. Lim , K. Rajaram , A. J. Bandodkar , S.‐W. Hwang , ACS Nano 2023, 17, 14822. 37497757 10.1021/acsnano.3c03063

[14] J.‐S. Shim , J. A. Rogers , S.‐K. Kang , Mater. Sci. Eng. R Rep. 2021, 145, 100624.

[15] Y. Gao , Y. Zhang , X. Wang , K. Sim , J. Liu , J. Chen , X. Feng , H. Xu , C. Yu , Sci. Adv. 2017, 3, 1701222. 10.1126/sciadv.1701222 PMC5580884 28879237

[16] J.‐W. Shin , D.‐J. Kim , T.‐M. Jang , W. B. Han , J. H. Lee , G.‐J. Ko , S. M. Yang , K. Rajaram , S. Han , H. Kang , J. H. Lim , C.‐H. Eom , A. J. Bandodkar , H. Min , S.‐W. Hwang , Nanomicro Lett. 2024, 16, 102. 38300387 10.1007/s40820-023-01268-2 PMC10834929

[17] Y. Wang , Z. Ma , W. He , Y. Zhang , P. Liu , Def. Technol. 2025, 44, 111.

[18] Y. Wang , C. Zhang , Z. Pang , Z. Ma , W. He , P. Liu , Y. Zhang , Chem. Eng. J. 2024, 500, 156412.

[19] A. Dutta , H. Cheng , Nanoscale 2023, 15, 4236. 36688506 10.1039/d2nr06068j

[20] E. Istif , M. Ali , E. Y. Ozuaciksoz , Y. Morova , L. Beker , ACS Omega 2024, 9, 2528. 38250408 10.1021/acsomega.3c07203 PMC10795112

[21] W. D. Chen , S.‐K. Kang , W. J. Stark , J. A. Rogers , R. N. Grass , Sens. Actuators, B 2019, 282, 52.

[22] A. Gumus , A. Alam , A. M. Hussain , K. Mishra , I. Wicaksono , G. A. Torres Sevilla , S. F. Shaikh , M. Diaz , S. Velling , M. T. Ghoneim , S. M. Ahmed , M. M. Hussain , Adv. Mater. Technol. 2017, 2, 1600264.

[23] S. Pandey , C. Mastrangelo , Micromachines 2022, 13, 242. 35208366 10.3390/mi13020242 PMC8877697

[24] S.‐K. Kang , S.‐W. Hwang , H. Cheng , S. Yu , B. H. Kim , J.‐H. Kim , Y. Huang , J. A. Rogers , Adv. Funct. Mater. 2014, 24, 4427.

[25] C. H. Lee , J.‐W. Jeong , Y. Liu , Y. Zhang , Y. Shi , S.‐K. Kang , J. Kim , J. S. Kim , N. Y. Lee , B. H. Kim , K.‐I. Jang , L. Yin , M. K. Kim , A. Banks , U. Paik , Y. Huang , J. A. Rogers , Adv. Funct. Mater. 2015, 25, 1338.

[26] M. D. Banea , L. F. M. da Silva , R. J. C. Carbas , R. D. S. G. Campilho , Int. J. Adhes. Adhes. 2014, 54, 191.

[27] N. Lai , M. Lv , H. Pan , Appl. Sci. 2023, 13, 7495.

[28] T.‐I. Lee , C. Kim , M. S. Kim , T.‐S. Kim , Polym. Test. 2016, 53, 70.

[29] F. Majid , M. Abbasi , Colloid Polym. Sci. 2016, 294, 1055.

[30] A. Olanrewaju , M. Beaugrand , M. Yafia , D. Juncker , Lab Chip 2018, 18, 2323. 30010168 10.1039/c8lc00458g

