# Supplementary material for: Stretchable and Biodegradable Thermally Expandable Composites with Microfluidics for On‐Demand and Programmable Destruction of Electronics
Source: Adv Sci (Weinh). 2025 Jun 30;12(36):e05487. doi: 10.1002/advs.202505487 (PMC12463090; doi:10.1002/advs.202505487)
Supplement: Supplementary file 1 — Supporting Information [file ADVS-12-e05487-s001.docx]

Supporting Information

Stretchable and biodegradable thermally expandable composites integrated with microfluidics for on-demand, programmable and selective destruction of electronic systems

Chan-Hwi Eom, Won Bae Han, Sungkeun Han, So Jeong Choi, Ikkyo Choi, Jeonguk Kim, Hyewon Cho, Li-Hyun Kim, Venkata Ramesh Naganaboina, Gwan-Jin Ko, Tae-Min Jang and Suk-Won Hwang*


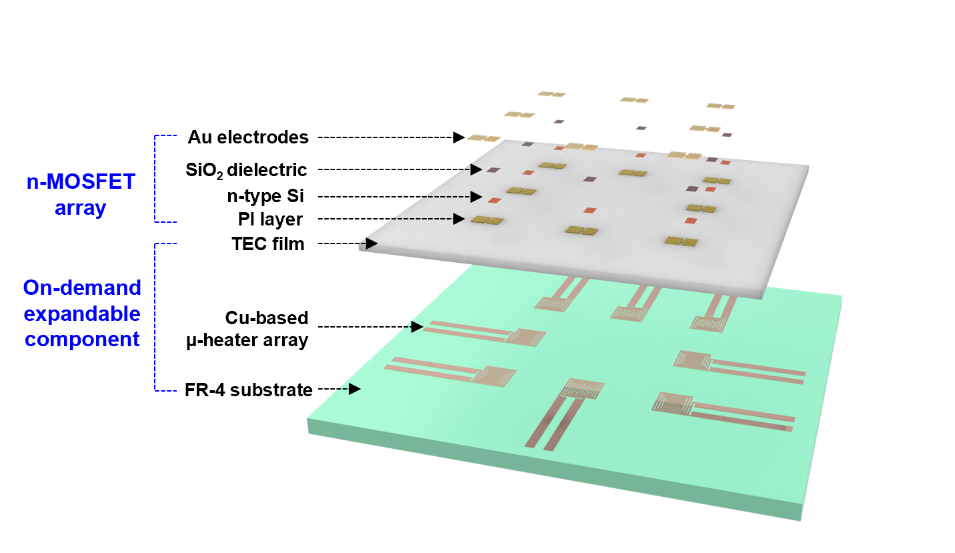


**Figure S1**. Exploded view of a flexible, destructive n-MOSFET array.


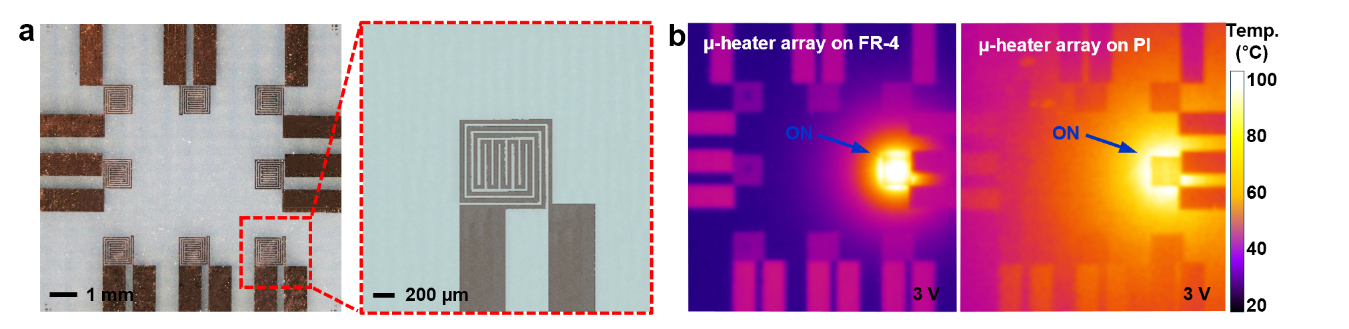


**Figure S2**. (a) Optical image (left) of a Cu foil-based μ-heater array with the magnified view (right). (b) Infrared (IR) images of the array formed on FR-4 and PI substrates while applying 3 V to a μ-heater. Compared to the PI substrate, FR-4 exhibited concentrated heat on the operating heater.


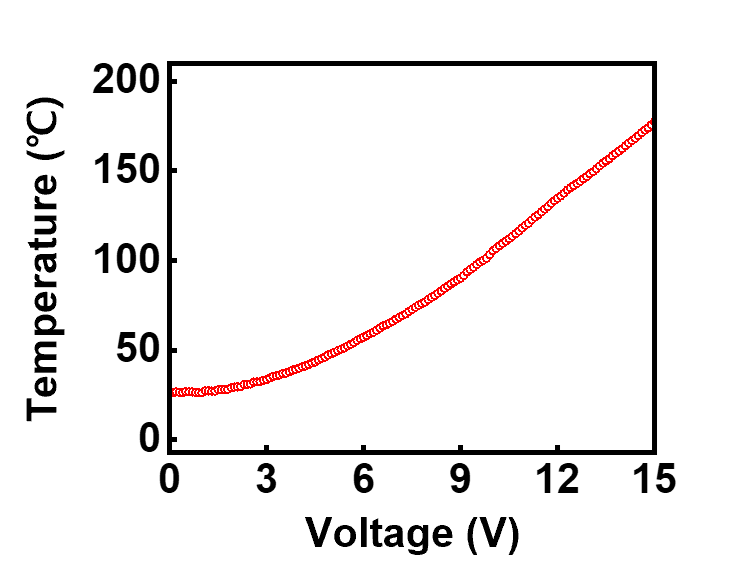


**Figure S3**. Voltage-temperature characteristics of a 200 nm thick Cu-based microheater (50 Ω).


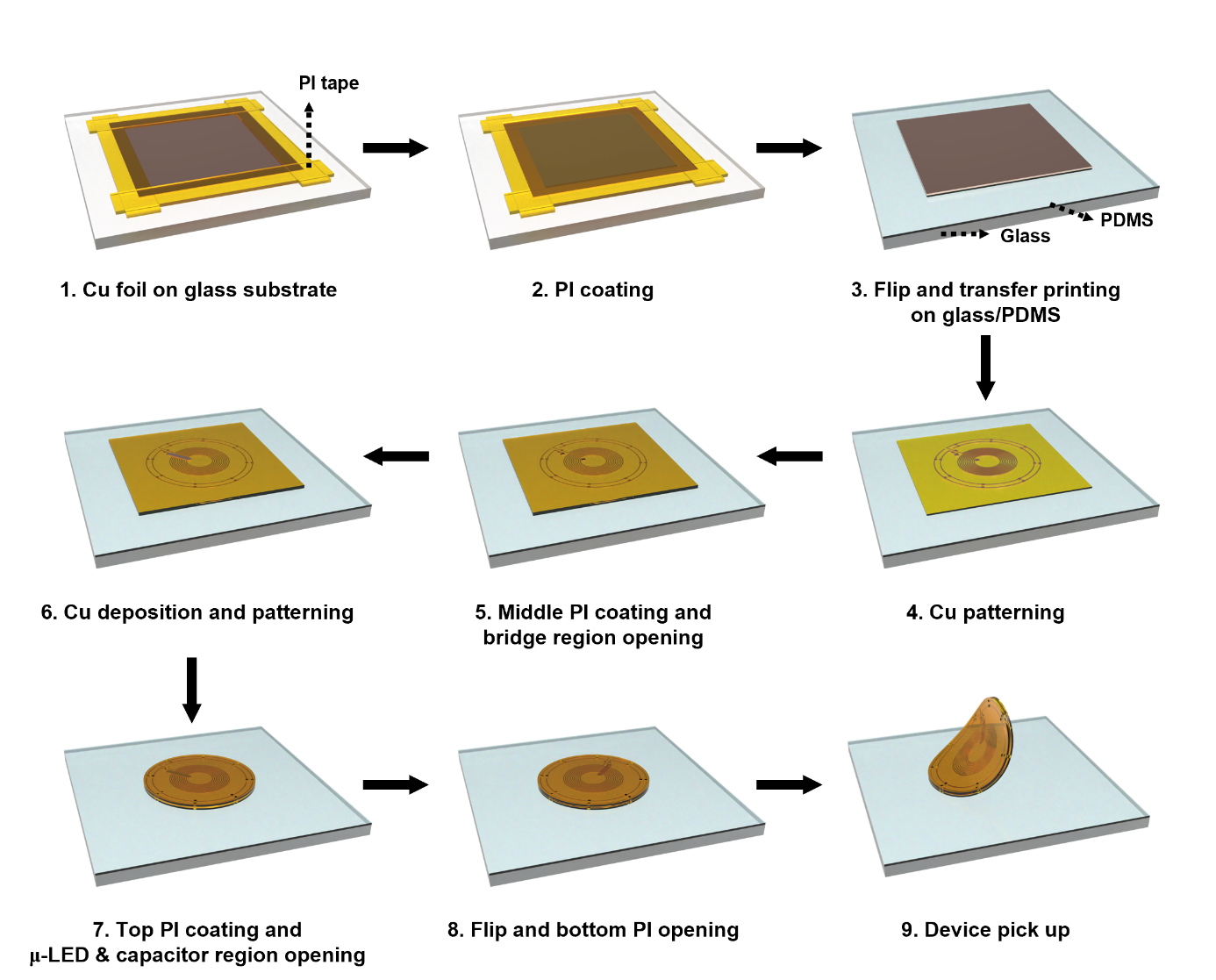


**Figure S4**. Fabrication process of a wireless, flexible optoelectronic system.


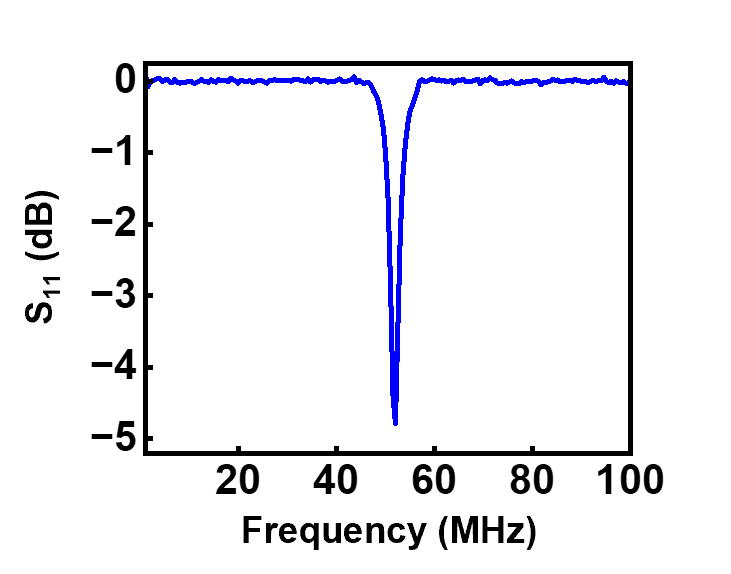


**Figure S5**. Measured resonant frequency of the optoelectronic device.


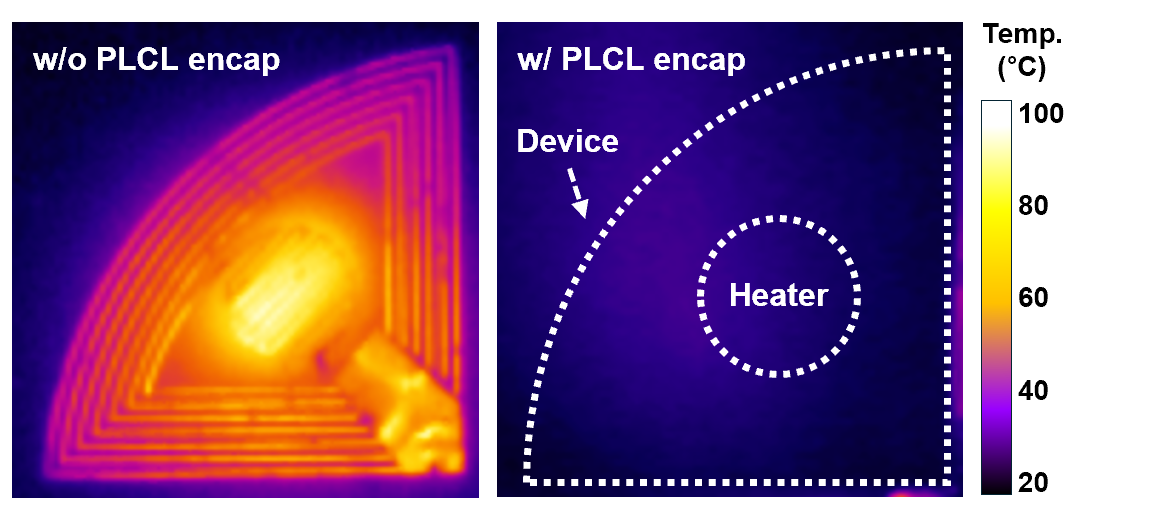


**Figure S6**. IR image of the drug delivery system during operation w/ and w/o PLCL encapsulation.


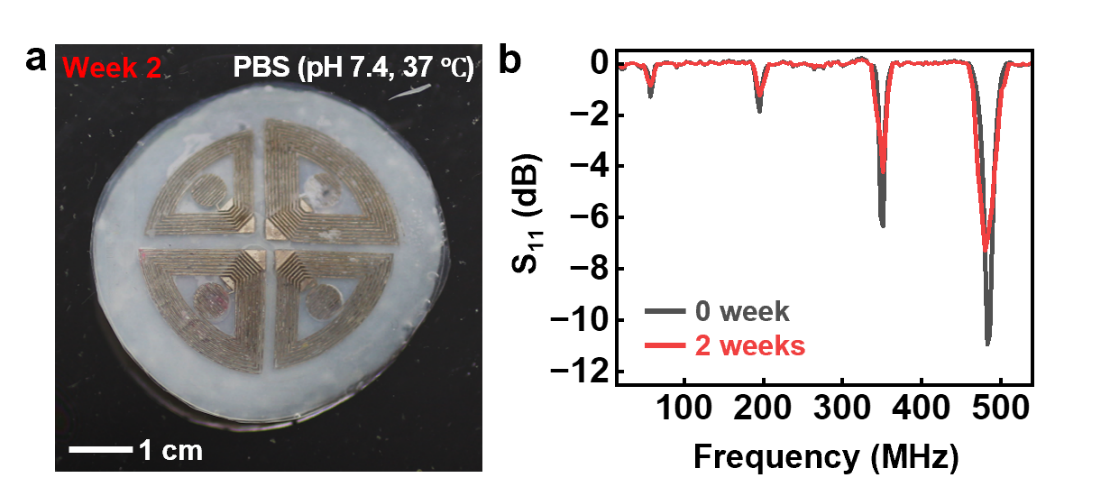


**Figure S7.** (a) Optical image and (b) measured resonant frequencies of the device two weeks after immersion in PBS (pH 7.4, 37 ^o^C).


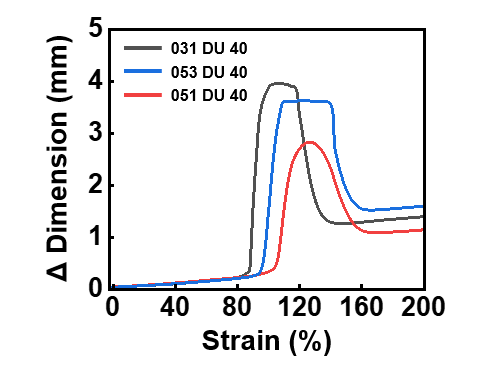


**Figure S8.** Coefficient of thermal expansion of various TEPs

**
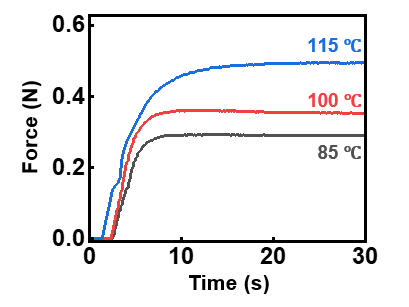
**

**Figure S9.** Temporal changes in thermal expansion forces of PLCL-based TEC film at different temperatures of 85 ℃, 100 ℃, and 115 ℃

**
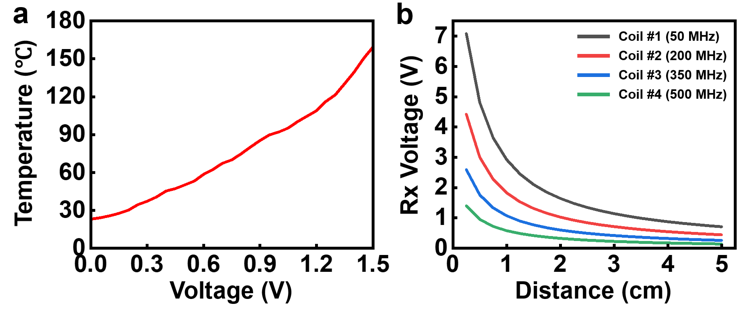
**

**Figure S10.** (a) Voltage–temperature calibration curves for microheater in drug-delivery system. (b) Induced voltage at each Rx coil measured over varying Tx–Rx distances.


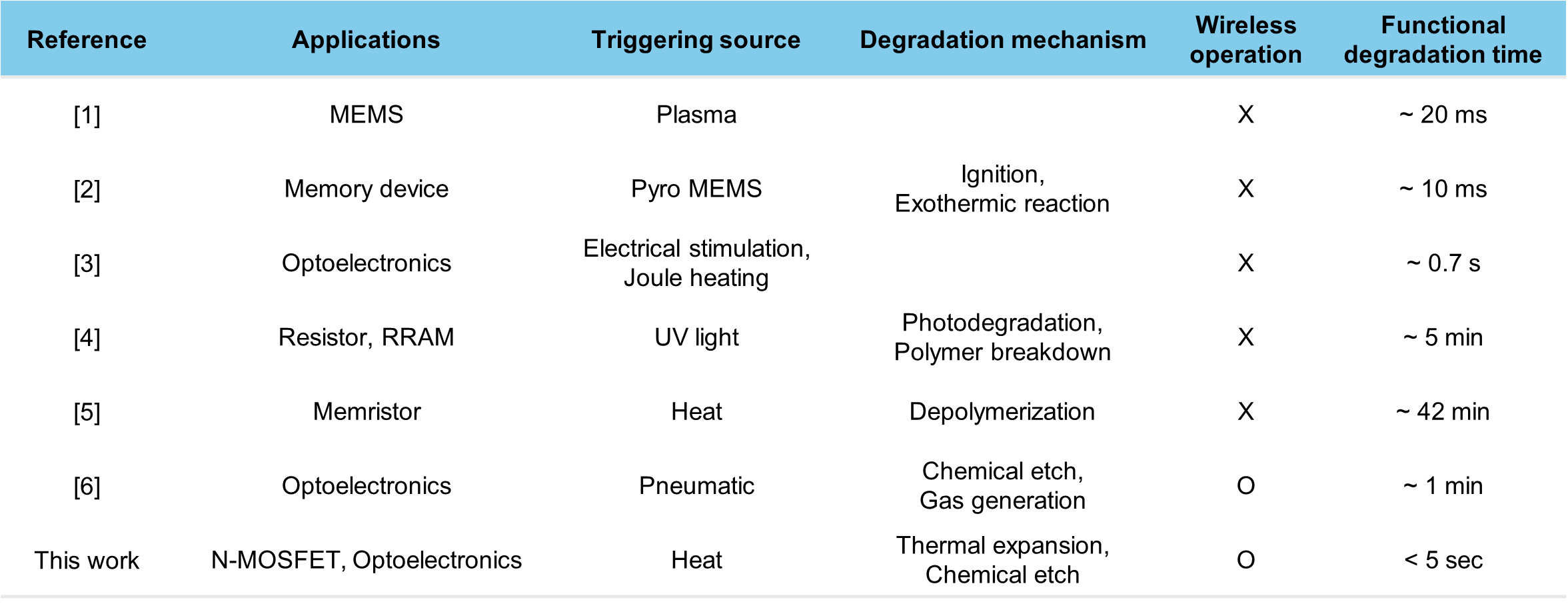


**Table S1**. Comparison of reported functional degradation systems and the proposed wireless TEC-based platform.

Supporting Reference

[1] Z. Zhang, J. Cheng, Y. Wang, F. Li, Y. Shen, C.-A. Wang, J. Xu, R. Shen, Y. Ye, *Chem. Eng. J*. **2023**, 451, 138361.

[2] F. Sevely, T. Wu, F. S. Ferreira de Sousa, L. Seguier, V. Brossa, S. Charlot, A. Esteve, C. Rossi, *Sens. Actuators A Phys*. **2022**, 346, 113838.

[3] Y. Wang, Z. Ma, P. Liu, W. He, *Chem. Eng. J*. **2023**, 473, 144981.

[4] S. Zhong, H. C. Wong, H. Y. Low, R. Zhao, *ACS Appl. Mater. Interfaces* **2021**, 13, 904.

[5] D. Liu, S. Zhang, H. Cheng, R. Peng, Z. Luo, *Sci. Rep*. **2019**, 9, 18107.

[6] J.-W. Shin, J. Chan Choe, J. H. Lee, W. B. Han, T.-M. Jang, G.-J. Ko, S. M. Yang, Y.-G. Kim, J. Joo, B. H. Lim, E. Park, S.-W. Hwang, *ACS Nano* **2021**, 15, 19310.
